# Supplementary material for: Potent immune-dependent anticancer effects of the non-cardiotoxic anthracycline aclarubicin
Source: Oncoimmunology. 2025 Jun 4;14(1):2515176. doi: 10.1080/2162402X.2025.2515176 (PMC12143712; doi:10.1080/2162402X.2025.2515176)
Supplement: REVISIONS_HIGHLIGHTS_Potent immune dependent anticancer effects of the non cardiotoxic anthracycline aclarubicin.docx [file KONI_A_2515176_SM9293.docx]

**Potent immune-dependent anticancer effects of the non-cardiotoxic anthracycline aclarubicin**

**Giulia Cerrato**^1,2,3,*,#^**, Allan Sauvat**^1,2,3,*^**, Mahmoud Abdellatif**^1,3,4^ **& Guido Kroemer**^1,2,3,5,#^

* Equally contributed

^#^ Corresponding authors

**Affiliations**

^1^Université Paris Cité, Sorbonne Université, Inserm, Centre de Recherche des Cordeliers, F-75006 Paris, France Centre de Recherche des Cordeliers, Equipe labellisée par la Ligue contre le cancer, Institut Universitaire de France, Paris, France

^2^Onco-Pheno-Screen Platform, Centre de Recherche des Cordeliers, Paris, France

^3^ Université Paris-Saclay, INSERM US23 / CNRS UAR 3655, Metabolomics and Cell Biology Platforms, Institut Gustave Roussy, Villejuif, France

^4^Department of Cardiology, Medical University of Graz, Graz, Austria

^5^Institut du Cancer Paris CARPEM, Department of Biology, Hôpital Européen Georges Pompidou, AP-HP, Paris, France

**Official email of all authors**

Giulia Cerrato: giulia.cerrato@gustaveroussy.fr

Allan Sauvat: allan.sauvat@gustaveroussy.fr

Mahmoud Abdellatif: mahmoud.abdellatif@medunigraz.at

Guido Kroemer: kroemer@orange.fr

**Abstract**

Aclarubicin (also called aclacinomycin A) is an antineoplastic from the anthracycline class that is used in China and Japan but not in Europe nor in the USA. Aclarubicin induces much less DNA damage than the classical anthracyclines doxorubicin, daunorubicin, epirubicin, idarubicin and the anthracene mitoxantrone, but is equally effective in inhibiting DNA-to-RNA transcription and in eliciting immunogenic stress in malignant cells. Accordingly, aclarubicin lacks the DNA damage-associated cardiotoxicity that is dose-limiting for classical anthracyclines. Conversely, aclarubicin is at least as potent as other anthracyclines in inducing immunogenic cell death (ICD), which is key for the mode of action of efficient chemotherapeutics. This combination of reduced toxicity and equivalent ICD-stimulatory activity may explain why, as compared to other anthracyclines, aclarubicin is particularly efficient against acute myeloid leukemia. As a result, we advocate for clinical studies seeking to replace the anthracyclines used in Western medicine by aclarubicin-like compounds. Such clinical studies should not only embrace hematological malignancies but should also concern solid cancers, including those in which ICD-inducing chemotherapies are followed by immunotherapies targeting the PD-1/PD-L1 interaction.

**Keywords**: Anticancer agents, integrated stress response, immune checkpoint inhibitors, immunosuppression, myelosuppression

**Introduction**

Historically, cancer chemotherapeutics or "cytotoxicants" were believed to mediate their antineoplastic effects by the induction of cytostasis (i.e., inhibition of proliferation) and cytolysis (i.e., killing of malignant cells)^1^. In mechanistic terms, such effects have been linked to cell biological responses to cytotoxic agents, in particular, cellular senescence (which results in a permanent arrest of the cell cycle)^2^ and regulated cell death through various subroutines such as apoptosis, ferroptosis and necroptosis^3^. Many chemotherapeutic agents bind to DNA or to proteins interacting with DNA and hence are considered as DNA-damaging agents^4^. Therefore, DNA damage has been considered as the prime mechanisms explaining the cytostatic and cytolytic effects of various anticancer agents including anthracyclines^5^. Indeed, most anthracyclines tend to enrich in the nuclei of cells, where they intercalate into DNA strands, thus inhibiting the DNA-interacting topoisomerase II, and thereby inducing double-stranded DNA breaks^6,7^.

In this context, it was thought that cancer chemotherapeutics generally mediate their effects through a direct (and exclusive) action on malignant cells and that anthracyclines - in particular - induce cancer cell killing through the induction of DNA damage^6,7^. In this view, the devastating side effects of anthracyclines including the rate-limiting cardiotoxicity causing long-term outcomes including premature aging and heart failure were thought to be mechanism-related and hence close-to-unavoidable. To manage such side effects, the cumulative dose of anthracyclines was limited by guidelines^8,9^. Moreover, one cardioprotective agent, dexrazoxane was approved by the FDA to reduce the cardiotoxicity of anthracyclines in infants^10^.

In this mini-review, we will argue that the aforementioned statements on the mode of action of chemotherapeutics and anthracyclines are inaccurate. First, the efficiency of cancer chemotherapeutics is dictated by their capacity of inducing antitumor immune responses subsequent to the induction of immunogenic stress and death of cancer cells rather than by the direct large-scale eradication of malignant cells. Second, the efficacy of anthracyclines does not rely on DNA damage but rather involves a DNA damage-independent inhibition of DNA-to-RNA transcription that then elicits the integrated stress response (ISR) favoring immunogenic cell death (ICD). Importantly, one particular anthracycline, aclarubicin (also called aclacinomycin A)^11^, which is only used in Asia, not in Europe nor the US, has the interesting property to combine the full potential of inducing ICD with the absence of DNA damage and cardiotoxicity.

**Direct cytotoxic *versus* indirect immunological anticancer effects of anthracyclines**

The view that anthracyclines mediate their anticancer effects by direct cytostatic or cytotoxic effects on malignant cells was challenged for the first time by mouse experiments showing that systemic chemotherapy with one single dose of doxorubicin against mouse colorectal cancers is only efficient in wild type mice bearing an intact immune system (in this case BALB/c mice bearing isogenic CT26 tumors), but not in immunodeficient *Foxn1*^nu^ mice lacking T lymphocytes^12^. Additional experiments revealed that cancer cells killed with anthracyclines (such as daunorubicin, doxorubicin) or the anthracene mitoxantrone *in vitro* were able to induce a protective anticancer immune response upon subcutaneous injection into histocompatible, immunocompetent mice, meaning that the vaccination prevented the growth of cancer cells that were inoculated into the opposite flank one week later ^12-14^. This phenomenon was referred to as "immunological cell death" and was demonstrated to obey strict rules with respect to the stress signals that had to be induced in cancer cells before they succumb to ICD, the danger-associated molecular patterns (DAMPs) associated with ICD, as well as the pattern recognition receptors (PRRs) involved in the recognition of such DAMPs, facilitating an effective immune response against dying cancer cells^15,16^. The exposure of the DAMP calreticulin on the cell surface, as well as the autophagy-dependent release of another DAMP, ATP, from cells depends on the integrated stress response (ISR) consisting in the phosphorylation of eukaryotic initiation factor 2α (eIF2α)^17^.

However, the most convincing results supporting the importance of anthracycline-induced antitumor immunity were obtained by correlative studies in cancer patients. First, it turned out that neoadjuvant anthracycline-based chemotherapy against breast cancer could be accurately predicted in its anatomopathological and clinical outcome by quantifying the density of tumor-infiltrating lymphocytes (TILs)^18^, suggesting that the pre-existing anticancer immune response determined therapeutic outcome. Later observations, demonstrated that anthracyclines also affected the cancer immune infiltrate in dynamic terms and that a chemotherapy-induced amelioration of the ratio of tumor-infiltrating cytotoxic T lymphocytes over regulatory T cells predicted the outcome of chemotherapy as well^19^. Even more convincingly, experimental studies in mice indicated that ICD-inducing chemotherapies could sensitize to subsequent immunotherapy with PD-1 blocking antibodies^20^, and clinical trials demonstrated that doxorubicin-based chemotherapy was particularly efficient in sensitizing triple-negative breast cancer^21^or leiomyosarcoma^22^ to subsequent PD-1 blockade.

In conclusion, experimental and clinical studies confirm the idea that anthracycline-based chemotherapies rely on anticancer immune responses to be fully efficient. Although anthracyclines undoubtedly mediate direct cytotoxic effects against cancer cells in vitro and in vivo, their long-term effects - beyond treatment discontinuatio - clearly involve an obligatory immune component.

**Anticancer effects of anthracyclines with and without DNA damage**

All classical anthracyclines induce massive DNA damage in cultured cancer cells, a phenomenon that has been ascribed to their capacity to inhibit topoisomerase 2 (TOP2). Indeed, TOP2 relieves torsional strain from DNA required for its replication or transcription by catalyzing two enzymatic reactions, first by introducing DNA double strand breaks (DSBs that allow unwinding of the DNA helix) and then by religating these DSBs to restore the integrity of DNA. Inhibition of this latter step by anthracyclines culminates in the accumulation of DSBs that spur the formation of microscopically-visible DNA damage foci reflecting the accumulation of DNA repair enzymes on the damaged DNA. If unresolved, DNA damage triggers stress signals that favor cellular senescence or cellular demise secondary to the activation of regulated cell death pathways^23^.

Singularly, among intercalating agents (**Fig.1A**), which are predominantly interfacial poisons, aclarubicin does not bind to TOP2^24^. Instead, it interferes with the initial catalytic step of TOP2 by preventing its association with DNA^23,25^, thus avoiding the formation of DSBs.

Moreover, through the inhibition of the DNA-TOP2 interaction, aclarubicin abolishes the induction of DSBs by both doxorubicin^26^ and etoposide^27^. Beyond this effect on TOP2, aclarubicin causes the degradation of RNA polymerase II^28^, in accord with a general inhibition of DNA-to-RNA transcription^29^. In addition, aclarubicin reduces the subnuclear mobility of TOP2, preventing its translocation to nucleoli in conditions of ATP depletion^27^. Nucleoli rapidly condense in cells treated with aclarubicin perhaps as a reflection of reduced RNA synthesis^29^. Aclarubicin and classical anthracyclines have similar effects on nucleolar condensation and inhibition of DNA-to-RNA transcription^29^. Moreover, aclarubicin and classical anthracyclines indistinguishably cause chromatin damage mediated by histone eviction from open chromosomal areas^30-32^ and inhibit the binding of nuclear factor kappa B (NF-κB) to DNA^33^. In *Drosophila* cells, chromatin changes induced by aclarubicin treatment are inhomogeneous in thus far that they are affected by promoter proximity and orientation^34^. However, it is not clear whether this phenomenon applies to mammalian cells and whether it differentiates aclarubicin from classical anthracyclines. Histone eviction can be clearly dissociated from TOP2 inhibition because specific TOP2 inhibitors such as etoposide lack the capacity of stimulating histone eviction^30^ and do not inhibit the transcription of NF-κB target genes^33^. This phenomenon could be explained by the capacity of the anthracyclines, but not etoposide, to stall DNA replication fork independently of TOP2^35^. Altogether, these observations are consistent with the morphological changes which occur in the nucleoli, as observed by transmission electron microscopy. Specifically, both doxorubicin and aclarubicin induce the dispersion of nucleolar substructures, namely the fibrillar center (FC), the dense fibrillar component (DFC) and the granular compartment (GC), whereas etoposide has only a minimal impact on nucleolar ultrastructure (**Fig. 1B**).

Importantly, the capacity of anthracyclines to induce ICD does not require the induction of DSB due to TOP2 inhibition. Thus, specific TOP2 inhibitors such as etoposide are lacking any capacity to elicit the stigmata of ICD *in vitro* (including the exposure or release of DAMPs such as calreticulin, adenosine triphosphate and high mobility G protein B1) and to elicit anticancer immune responses *in vivo*^12,36^. In stark contrast, aclarubicin is as efficient as classical anthracyclines in eliciting ICD^29^, commensurate with its capacity to inhibit DNA-to-RNA transcription, which is sufficient to elicit nucleolar condensation (a novel morphological hallmark of ICD)^17,29^ (**Fig. 2**). Beyond its capacity to induce the stigmata of ICD, aclarubicin has been shown to stimulate the production of effector molecules by natural killer (NK) cells, as documented for perforin and granzyme B, and enhances the NK-mediated killing of allogeneic acute myeloid leukemia cells *in vitro*^37^.

Altogether, compelling evidence supports the notion that the immune-related anticancer effects of anthracyclines do not rely on the induction of DNA damage in the form of DSBs, but more likely on DNA replication stress.

**Aclarubicin, an anthracycline lacking cardiotoxicity**

Cardiotoxicity induced by the classical anthracyclines doxorubicin, daunorubicin, epirubicin, idarubicin and mitoxantrone is dose-limiting, meaning that the cumulative dose of these drugs is limited by the FDA and EMA to avoid lethal cardiomyopathy^8,9^. Such anthracyclines often cannot be used against recurring tumors even though they could still be effective. The mechanism of cardiotoxicity involves premature cardiac aging due to cardiomyocyte senescence^38^. Dexrazoxane, an iron chelator, has been approved for intravenous administration together with anthracyclines to reduce their toxicity^10^. However, the clinical use of dexrazoxane remains limited and continues to undergo scrutiny in clinical trials^39,40^.

In sharp contrast to classical anthracyclines, aclarubicin and an N,N-dimethyl derivative of doxorubicin, which both induce histone eviction but do not induce DSBs, lack cardiotoxicity^29^. Similarly, amrubicin, which induces DSBs but not histone eviction^31^ has no cardiotoxic activity^41^. Outside of the class of anthracyclines, BMH21, a specific inhibitor of DNA-to-RNA transcription that induces histone eviction without DSBs^29^, as well as specific TOP2 inhibitors such as etoposide and teniposide, which massively induce DSBs but no histone eviction, completely lack cardiotoxicity^29,42,43^. These observations are compatible with the hypothesis that cardiotoxicity mediated by classical anthracyclines is due to a combination of two effects, namely histone eviction plus DNA damage, and that either of these two phenomena alone is insufficient to cause premature cardiac aging^44^.

In accord with the fact that aclarubicin is not cardiotoxic, it can be safely administered to cancer patients even after the maximum cumulative dose of either doxorubicin or idarubicin has been reached^45^. Importantly, a side-by-side comparison of different chemotherapeutic agents led to the conclusion that aclarubicin is also inducing much less secondary cancers than does doxorubicin and etoposide in mice^31^. Other side effects that appear to be attenuated for aclarubicin compared to doxorubicin are alopecia^46^. In contrast, aclarubicin induces a similar degree of myelosuppression to classical anthracyclines^46^.

Altogether, these findings indicate that aclarubicin has a much better safety profile than classical anthracyclines.

**Comparison of the clinical efficacy of aclarubicin and classical anthracyclines**

When injected into mice, the biodistribution of aclarubicin is different from that of daunorubicin and idarubicin. Aclarubicin reaches high concentrations in lymphoid organs (spleen, thymus and lymph nodes) similar to daunorubicin and idarubicin but lower concentrations (by approximately one log) in lung, kidney, liver, heart and plasma^45^.

This matches the preferential use of aclarubicin against hematological cancers, in particular acute myeloid leukemia in the countries where it is regularly approved, namely, China and Japan. Indeed, the so-called CAG regimen, composed by low-dose cytarabine, aclarubicin and recombinant granulocyte-colony stimulating factor (G-CSF), is been widely used in China and Japan for the treatment of AML^47,48^. A single-center retrospective study compared the efficacy of CAG to other salvage chemotherapies in relapsed/refractory acute myeloid leukemia (r/rAML) patients to discover that the aclarubicin-containing CAG regimen assured a notable 23% increase in 5-year overall survival compared to other intensive chemotherapies^45^.

Thus far, no clinical trials have compared the outcome of AML treatments with different anthracyclines including aclarubicin. Moreover, the literature of aclarubicin effects on solid cancers is scarce (without any publications since 2000), rendering a direct comparison of aclarubicin with other anthracyclines difficult.

**Conclusions**

In synthesis, emerging evidence challenges the traditional view that anthracyclines exert anticancer effects solely through direct cytotoxicity and DNA damage. Instead, their efficacy relies significantly on inducing ICD, which results in antitumor immune responses that prolong the anticancer effects of anthracyclines beyond treatment discontinuation. Notably, aclarubicin distinguishes itself from classical anthracyclines by effectively inducing ICD without causing DNA double-strand breaks, thus avoiding the cardiotoxicity that limits conventional anthracycline use. This unique property allows aclarubicin to be administered beyond standard cumulative dose limits and offers a safer alternative for patients with hematological malignancies. However, despite its promising therapeutic profile, aclarubicin remains underutilized outside Asia, and further clinical studies are needed to fully assess its potential across a broader range of cancers, including solid tumors. In this context, it will be particularly interesting to test combination regimens combining aclarubicin with immunotherapy targeting PD-1 or PD-L1.

**Acknowledgements**

Electron microscopy studies were conducted by Sylvie Souquère et Gérard Pierron at the AMMICA-UAR3655 electron microscopy facility of the Institute Gustave Roussy (IGR) in Villejuif, France.

**Funding details**

GK is supported by the Ligue contre le Cancer (équipe labellisée); Agence Nationale de la Recherche (ANR-22-CE14-0066 VIVORUSH, ANR-23-CE44-0030 COPPERMAC, ANR-23-R4HC-0006 Ener-LIGHT); Association pour la recherche sur le cancer (ARC); Cancéropôle Ile-de-France; Fondation pour la Recherche Médicale (FRM); a donation by Elior; European Joint Programme on Rare Diseases (EJPRD) Wilsonmed; European Research Council Advanced Investigator Award (ERC-2021-ADG, Grant No. 101052444; project acronym: ICD-Cancer, project title: Immunogenic cell death (ICD) in the cancer-immune dialogue); The ERA4 Health CardInnov Grant Ener-LIGHT; European Union Horizon 2020 research and innovation programmes Oncobiome (grant agreement number: 825410, Project Acronym: ONCOBIOME, Project title: Gut OncoMicrobiome Signatures [GOMS] associated with cancer incidence, prognosis and prediction of treatment response, Prevalung (grant agreement number 101095604, Project Acronym: PREVALUNG EU, project title: Biomarkers affecting the transition from cardiovascular disease to lung cancer: towards stratified interception), Neutrocure (grant agreement number 861878 : Project Acronym: Neutrocure ; project title: Development of “smart” amplifiers of reactive oxygen species specific to aberrant polymorphonuclear neutrophils for treatment of inflammatory and autoimmune diseases, cancer and myeloablation); National support managed by the Agence Nationale de la Recherche under the France 2030 programme (reference number 21-ESRE-0028, ESR/Equipex+ Onco-Pheno-Screen); Hevolution Network on Senescence in Aging (reference HF-E Einstein Network); Institut National du Cancer (INCa); Institut Universitaire de France; LabEx Immuno-Oncology ANR-18-IDEX-0001; a Cancer Research ASPIRE Award from the Mark Foundation; PAIR-Obésité INCa_1873, the RHUs Immunolife and LUCA-pi (ANR-21-RHUS-0017 and ANR-23-RHUS-0010, both dedicated to France Relance 2030); Seerave Foundation; SIRIC Cancer Research and Personalized Medicine (CARPEM, SIRIC CARPEM INCa-DGOS-Inserm-ITMO Cancer_18006 supported by Institut National du Cancer, Ministère des Solidarités et de la Santé and INSERM). This study contributes to the IdEx Université de Paris Cité ANR-18-IDEX-0001. MA acknowledges support from the Medical University of Graz (Flagship Project VASCHEALTH), BioTechMed-Graz (Young Researcher Group), and the Austrian Science Fund (FWF; DOI: https://doi.org/10.55776/P34926). MA and GK received additional funding from FWF (DOI: https://doi.org/10.55776/I6931) and ANR under the umbrella of the Partnership Fostering a European Research Area for Health (ERA4Health) (GA N° 101095426 of the EU Horizon Europe Research and Innovation Programme (Ener-LIGHT consortium).

**Disclosure statement**

GK has been holding research contracts with Daiichi Sankyo, Eleor, Kaleido, Lytix Pharma, PharmaMar, Osasuna Therapeutics, Samsara Therapeutics, Sanofi, Sutro, Tollys, and Vascage. GK is on the Board of Directors of the Bristol Myers Squibb Foundation France. GK is a scientific co-founder of everImmune, Osasuna Therapeutics, Samsara Therapeutics and Therafast Bio. GK is in the scientific advisory boards of Hevolution, Institut Servier, Longevity Vision Funds and Rejuveron Life Sciences. GK is the inventor of patents covering therapeutic targeting of aging, cancer, cystic fibrosis and metabolic disorders. GK’s wife, Laurence Zitvogel, has held research contracts with Glaxo Smyth Kline, Incyte, Lytix, Kaleido, Innovate Pharma, Daiichi Sankyo, Pilege, Merus, Transgene, 9 m, Tusk and Roche, was on the on the Board of Directors of Transgene, is a cofounder of everImmune, and holds patents covering the treatment of cancer and the therapeutic manipulation of the microbiota. GK’s brother, Romano Kroemer, was an employee of Sanofi and now consults for Boehringer-Ingelheim. The funders had no role in the design of the study; in the writing of the manuscript, or in the decision to publish the results. No disclosures were reported by the other authors.

**CRediT Roles**

Funding acquisition: G.K., Visualization: G.C., A.S., Writing – original draft: G.K., A.S., G.C., Writing – review and editing: M.A.

**Data availability statement**

The authors confirm that the data supporting the findings of this study are available within the article.

**References**

1 Hanahan, D. & Weinberg, R. A. Hallmarks of cancer: the next generation. *Cell* **144**, 646-674, doi:10.1016/j.cell.2011.02.013 (2011).

2 Galluzzi, L. *et al.* Systems biology of cisplatin resistance: past, present and future. *Cell Death Dis* **5**, e1257, doi:10.1038/cddis.2013.428 (2014).

3 Galluzzi, L. *et al.* Molecular mechanisms of cell death: recommendations of the Nomenclature Committee on Cell Death 2018. *Cell Death Differ* **25**, 486-541, doi:10.1038/s41418-017-0012-4 (2018).

4 Ng, Y. B. & Akincilar, S. C. Shaping DNA damage responses: Therapeutic potential of targeting telomeric proteins and DNA repair factors in cancer. *Curr Opin Pharmacol* **76**, 102460, doi:10.1016/j.coph.2024.102460 (2024).

5 Cummings, J., Bartoszek, A. & Smyth, J. F. Determination of covalent binding to intact DNA, RNA, and oligonucleotides by intercalating anticancer drugs using high-performance liquid chromatography. Studies with doxorubicin and NADPH cytochrome P-450 reductase. *Anal Biochem* **194**, 146-155, doi:10.1016/0003-2697(91)90162-m (1991).

6 Spencer, D. M. *et al.* DNA repair in response to anthracycline-DNA adducts: a role for both homologous recombination and nucleotide excision repair. *Mutat Res* **638**, 110-121, doi:10.1016/j.mrfmmm.2007.09.005 (2008).

7 Saffi, J. *et al.* Effect of the anti-neoplastic drug doxorubicin on XPD-mutated DNA repair-deficient human cells. *DNA Repair (Amst)* **9**, 40-47, doi:10.1016/j.dnarep.2009.10.003 (2010).

8 Lotrionte, M. *et al.* Review and meta-analysis of incidence and clinical predictors of anthracycline cardiotoxicity. *Am J Cardiol* **112**, 1980-1984, doi:10.1016/j.amjcard.2013.08.026 (2013).

9 Sadurska, E. Current Views on Anthracycline Cardiotoxicity in Childhood Cancer Survivors. *Pediatr Cardiol* **36**, 1112-1119, doi:10.1007/s00246-015-1176-7 (2015).

10 Sayed, A. *et al.* Long-term effectiveness of empiric cardio-protection in patients receiving cardiotoxic chemotherapies: A systematic review & bayesian network meta-analysis. *Eur J Cancer* **169**, 82-92, doi:10.1016/j.ejca.2022.03.024 (2022).

11 Murzyn, A. *et al.* Aclarubicin: contemporary insights into its mechanism of action, toxicity, pharmacokinetics, and clinical standing. *Cancer Chemother Pharmacol* **94**, 123-139, doi:10.1007/s00280-024-04693-1 (2024).

12 Casares, N. *et al.* Caspase-dependent immunogenicity of doxorubicin-induced tumor cell death. *J Exp Med* **202**, 1691-1701, doi:10.1084/jem.20050915 (2005).

13 Panaretakis, T. *et al.* The co-translocation of ERp57 and calreticulin determines the immunogenicity of cell death. *Cell Death Differ* **15**, 1499-1509, doi:10.1038/cdd.2008.67 (2008).

14 Ma, Y. *et al.* Contribution of IL-17-producing gamma delta T cells to the efficacy of anticancer chemotherapy. *J Exp Med* **208**, 491-503, doi:10.1084/jem.20100269 (2011).

15 Galluzzi, L., Guilbaud, E., Schmidt, D., Kroemer, G. & Marincola, F. M. Targeting immunogenic cell stress and death for cancer therapy. *Nat Rev Drug Discov* **23**, 445-460, doi:10.1038/s41573-024-00920-9 (2024).

16 Kroemer, G., Montegut, L., Kepp, O. & Zitvogel, L. The danger theory of immunity revisited. *Nat Rev Immunol* **24**, 912-928, doi:10.1038/s41577-024-01102-9 (2024).

17 Humeau, J. *et al.* Inhibition of transcription by dactinomycin reveals a new characteristic of immunogenic cell stress. *EMBO Mol Med* **12**, e11622, doi:10.15252/emmm.201911622 (2020).

18 Denkert, C. *et al.* Tumour-infiltrating lymphocytes and prognosis in different subtypes of breast cancer: a pooled analysis of 3771 patients treated with neoadjuvant therapy. *Lancet Oncol* **19**, 40-50, doi:10.1016/S1470-2045(17)30904-X (2018).

19 Senovilla, L. *et al.* An immunosurveillance mechanism controls cancer cell ploidy. *Science* **337**, 1678-1684, doi:10.1126/science.1224922 (2012).

20 Pfirschke, C. *et al.* Immunogenic Chemotherapy Sensitizes Tumors to Checkpoint Blockade Therapy. *Immunity* **44**, 343-354, doi:10.1016/j.immuni.2015.11.024 (2016).

21 Voorwerk, L. *et al.* Immune induction strategies in metastatic triple-negative breast cancer to enhance the sensitivity to PD-1 blockade: the TONIC trial. *Nat Med* **25**, 920-928, doi:10.1038/s41591-019-0432-4 (2019).

22 Martin-Broto, J. *et al.* Phase Ib Study for the Combination of Doxorubicin, Dacarbazine, and Nivolumab as the Upfront Treatment in Patients With Advanced Leiomyosarcoma: A Study by the Spanish Sarcoma Group (GEIS). *J Clin Oncol* **43**, 297-307, doi:10.1200/JCO.24.00358 (2025).

23 Nitiss, J. L. Targeting DNA topoisomerase II in cancer chemotherapy. *Nat Rev Cancer* **9**, 338-350, doi:10.1038/nrc2607 (2009).

24 Sorensen, B. S. *et al.* Mode of action of topoisomerase II-targeting agents at a specific DNA sequence. Uncoupling the DNA binding, cleavage and religation events. *J Mol Biol* **228**, 778-786, doi:10.1016/0022-2836(92)90863-f (1992).

25 Sehested, M. & Jensen, P. B. Mapping of DNA topoisomerase II poisons (etoposide, clerocidin) and catalytic inhibitors (aclarubicin, ICRF-187) to four distinct steps in the topoisomerase II catalytic cycle. *Biochem Pharmacol* **51**, 879-886, doi:10.1016/0006-2952(95)02241-4 (1996).

26 Gajek, A., Rogalska, A. & Koceva-Chyla, A. Aclarubicin in subtoxic doses reduces doxorubicin cytotoxicity in human non-small cell lung adenocarcinoma (A549) and human hepatocellular carcinoma (HepG2) cells by decreasing DNA damage. *Toxicol In Vitro* **55**, 140-150, doi:10.1016/j.tiv.2018.12.015 (2019).

27 Morotomi-Yano, K. & Yano, K. I. Aclarubicin Reduces the Nuclear Mobility of Human DNA Topoisomerase IIbeta. *Int J Mol Sci* **25**, doi:10.3390/ijms251910681 (2024).

28 Espinoza, J. A. *et al.* Chromatin damage generated by DNA intercalators leads to degradation of RNA Polymerase II. *Nucleic Acids Res* **52**, 4151-4166, doi:10.1093/nar/gkae069 (2024).

29 Cerrato, G. *et al.* AI-based classification of anticancer drugs reveals nucleolar condensation as a predictor of immunogenicity. *Mol Cancer* **23**, 275, doi:10.1186/s12943-024-02189-3 (2024).

30 Pang, B. *et al.* Drug-induced histone eviction from open chromatin contributes to the chemotherapeutic effects of doxorubicin. *Nat Commun* **4**, 1908, doi:10.1038/ncomms2921 (2013).

31 Qiao, X. *et al.* Uncoupling DNA damage from chromatin damage to detoxify doxorubicin. *Proc Natl Acad Sci U S A* **117**, 15182-15192, doi:10.1073/pnas.1922072117 (2020).

32 Neefjes, J., Gurova, K., Sarthy, J., Szabo, G. & Henikoff, S. Chromatin as an old and new anticancer target. *Trends Cancer* **10**, 696-707, doi:10.1016/j.trecan.2024.05.005 (2024).

33 Chora, A. F. *et al.* DNA damage independent inhibition of NF-kappaB transcription by anthracyclines. *Elife* **11**, doi:10.7554/eLife.77443 (2022).

34 Wooten, M., Takushi, B., Ahmad, K. & Henikoff, S. Aclarubicin stimulates RNA polymerase II elongation at closely spaced divergent promoters. *Sci Adv* **9**, eadg3257, doi:10.1126/sciadv.adg3257 (2023).

35 Van Ravenstein, S. X. *et al.* Topoisomerase II poisons inhibit vertebrate DNA replication through distinct mechanisms. *EMBO J* **41**, e110632, doi:10.15252/embj.2022110632 (2022).

36 Obeid, M. *et al.* Ecto-calreticulin in immunogenic chemotherapy. *Immunol Rev* **220**, 22-34, doi:10.1111/j.1600-065X.2007.00567.x (2007).

37 Ye, Y. *et al.* Aclacinomycin enhances the killing effect of allogeneic NK cells on acute myeloid leukemia cells by inducing immunogenic cell death. *Front Immunol* **16**, 1521939, doi:10.3389/fimmu.2025.1521939 (2025).

38 Montegut, L. *et al.* High plasma concentrations of acyl-coenzyme A binding protein (ACBP) predispose to cardiovascular disease: Evidence for a phylogenetically conserved proaging function of ACBP. *Aging Cell* **22**, e13751, doi:10.1111/acel.13751 (2023).

39 Upshaw, J. N. *et al.* Dexrazoxane to Prevent Cardiotoxicity in Adults Treated with Anthracyclines: JACC: CardioOncology Controversies in Cardio-Oncology. *JACC CardioOncol* **6**, 322-324, doi:10.1016/j.jaccao.2024.02.004 (2024).

40 Linschoten, M. *et al.* Rationale and design of the HOVON 170 DLBCL-ANTICIPATE trial: preventing anthracycline-induced cardiac dysfunction with dexrazoxane. *Cardiooncology* **11**, 8, doi:10.1186/s40959-025-00303-y (2025).

41 Matsukida, A. *et al.* Efficacy and Safety of Amrubicin Monotherapy After Chemoradiotherapy in Patients With Relapsed Limited Disease Small-cell Lung Cancer. *Anticancer Res* **45**, 733-741, doi:10.21873/anticanres.17461 (2025).

42 Pai, V. B. & Nahata, M. C. Cardiotoxicity of chemotherapeutic agents: incidence, treatment and prevention. *Drug Saf* **22**, 263-302, doi:10.2165/00002018-200022040-00002 (2000).

43 Tinajero, J., Ngo, D., Salhotra, A. & Koller, P. Cladribine, Cytarabine, and Etoposide-Based Regimens Are Safe and Tolerable In Relapsed and Refractory Acute Myeloid Leukemia Patients. *Acta Haematol* **146**, 540-545, doi:10.1159/000534024 (2023).

44 van Gelder, M. A. *et al.* Re-Exploring the Anthracycline Chemical Space for Better Anti-Cancer Compounds. *J Med Chem* **66**, 11390-11398, doi:10.1021/acs.jmedchem.3c00853 (2023).

45 Qiao, X. *et al.* Diversifying the anthracycline class of anti-cancer drugs identifies aclarubicin for superior survival of acute myeloid leukemia patients. *Mol Cancer* **23**, 120, doi:10.1186/s12943-024-02034-7 (2024).

46 Rothig, H. J., Kraemer, H. P. & Sedlacek, H. H. Aclarubicin: experimental and clinical experience. *Drugs Exp Clin Res* **11**, 123-125 (1985).

47 Yamada, K. *et al.* Concurrent use of granulocyte colony-stimulating factor with low-dose cytosine arabinoside and aclarubicin for previously treated acute myelogenous leukemia: a pilot study. *Leukemia* **9**, 10-14 (1995).

48 Wei, G. *et al.* A meta-analysis of CAG (cytarabine, aclarubicin, G-CSF) regimen for the treatment of 1029 patients with acute myeloid leukemia and myelodysplastic syndrome. *J Hematol Oncol* **4**, 46, doi:10.1186/1756-8722-4-46 (2011).

49 O'Boyle, N. M. *et al.* Open Babel: An open chemical toolbox. *J Cheminform* **3**, 33, doi:10.1186/1758-2946-3-33 (2011).

50 Ruiz-Carmona, S. *et al.* rDock: a fast, versatile and open source program for docking ligands to proteins and nucleic acids. *PLoS Comput Biol* **10**, e1003571, doi:10.1371/journal.pcbi.1003571 (2014).

**Legends to Figures**

**Figure 1. DNA intercalation and consequences on nuclear morphology**

**(A)** Ligand 3D structures from typical intercalating agents were generated from SMILES using Open Babel toolbox^49^ ([http://openbabel.org](https://openbabel.org/)) and receptor structure (DNA) was retrieved from Protein Data Bank (PDB) (209D) (<https://www.rcsb.org>). Docking simulations performed using rDock program^50^ are displayed. **(B)** Ultra-thin sections of fixed human osteosarcoma U2OS cells left untreated (control) or treated with 5 μM aclarubicin, 10 μM doxorubicin, 10 μM etoposide for 4 hours were stained with uranyl acetate and lead citrate and then acquired using electron microscopy. Representative micrographs are shown. Arrows indicate the presence of fibrillar centers (FC), granular components (GC) and dense fibrillar component (DFC). Scale bar equals 1 μm.

**Figure 2. Divergent mechanisms and toxicities of DNA- and chromatin-damaging anticancer drugs**

Classical anthracyclines including doxorubicin, daunorubicin, idarubicin and the anthracene mitoxantrone are known for their ability to damage both DNA - by poisoning topoisomerase 2 (TOP2) and inducing double-stranded DNA breaks (DSBs) - and chromatin, through histone eviction. The downstream inhibition of DNA-to-RNA transcription triggers immunogenic cell death (ICD). While these compounds are potent anticancer agents, their dual activity also causes side effects such as cardiotoxicity and secondary cancers. In contrast, aclarubicin specifically induces chromatin damage, maintaining strong anticancer efficacy but fewer associated toxic effects. Conversely, amrubicin and etoposide induce DSBs without causing histone eviction, and are not associated with cardiotoxicity.
