# Supplementary material for: Potent immune-dependent anticancer effects of the non-cardiotoxic anthracycline aclarubicin
Source: Oncoimmunology. 2025 Jun 4;14(1):2515176. doi: 10.1080/2162402X.2025.2515176 (PMC12143712; doi:10.1080/2162402X.2025.2515176)
Supplement: Point by point reply.docx [file KONI_A_2515176_SM9294.docx]

Oncoimmunology Ms. No. 250158741

First author: Dr. Cerrato

Revision date: May 20, 2025

**Point-by-Point Reply to Reviewer No. 1**

**General comments by Reviewer No. 1**: Cerrato and colleagues provide a compelling manuscript calling for increased substitution of existing anthracycline medicines in Western healthcare systems with Aclarubicin. The text is sound, the arguments compelling, and the mechanisms of action of the drug illustrated. I recommend publication.

**Our response: We thank the Reviewer for her/his positive evaluation of our paper.**

**Specific comment No. 1 by Reviewer No. 1**: If the authors wish, they could consider adding a third figure on a concrete potential (combination) treatment scheme, which could serve as a template or starting point for future clinical investigations.

**Our response: We refrained from adding a third figure outlinging possible treatment schemes involving aclarabicin. Indeed, the second reviewer asked us to tone down the conclusions of our paper, and inclusing of a figure of this kind might be evaluated as a strong statement in favor of the utility of our hypothesis. Instead, we added a phrase to the Concluding Remarks of the paper (page 11) stating that "** **In this context, it will be particularly interesting to test combination regimens combining aclarubicin with immunotherapy targeting PD-1 or PD-L1."**

**Specific comment No. 1 by Reviewer No. 1**: In addition, information from a recent article could be included describing additional effects on RNA polymerase II of aclarubicin that have not been mentioned yet (10.1126/sciadv.adg3257).

**Our response: The Reviewer refers to PMID: 37315134 (Wooten et al. Sci Adv. 2023 Jun 16;9(24):eadg3257). We have cited this paper, which shows that - in *Drosophila* cells - chromatin changes induced by aclarubicin treatment are inhomogeneous in thus far that they are affected by promoter proximity and orientation (page 7).**

Oncoimmunology Ms. No. 250158741

First author: Dr. Cerrato

Revision date: May 20, 2025

**Point-by-Point Reply to Reviewer No. 2**

**General comments by Reviewer No. 2**: This comprehensive review presents a novel perspective on the mechanism of action of anthracyclines. The manuscript is well-written and informative.

**Our response: We thank the Reviewer for stating that our review is comprehensive and informative.**

**Specific comment by Reviewer No. 2**: While I agree that recent studies question the association between DNA damage induction and the efficacy of anthracyclines, the authors claim that the primary mechanism of anthracyclines is related to immune system modulation. I acknowledge that modulation of the immune response is indeed a component of anthracyclines' activity. However, in my opinion, the evidence provided is not sufficient to support the assertion that this is their main mode of action.

Anthracyclines (including aclarubicin) demonstrate strong activity in vitro, suggesting that their effects may be, at least in part, independent of the immune system.

Therefore, I recommend softening the statements regarding the significance of immune response in the mechanism of action of anthracyclines.

Also, the hypothesis that DNA-to-RNA transcription inhibition is the main driver of anthracycline efficacy is intriguing, but the manuscript presents it too strongly and should instead frame it more cautiously as a hypothesis.

**Our response: The Reviewer asks us to soften the statement that anthracycline effects are mostly mediated by immune responses. We have edited the text to make clear that anthracyclines do have direct cytotoxic effects measurable in vitro and in vivo, but that their long-term effects (beyond treatment discontinuation) clearly involve an obligatory immune component (page 6).**

**The Referee also asked us to make clear that we formulate a hypothesis (rather than a certitude) that DNA-to-RNA transcription inhibition accounts for the therapeutic efficacy of anthracyclines. We have done so in the revised version of our paper by stating that the evidence supports the notion that the immune-related anticancer effects of anthracyclines do not rely on the induction of DNA damage in the form of DSBs, but more likely on DNA replication stress (page 8).**
